# Data-Driven Automated Cardiac Health Management with Robust Edge Analytics and De-Risking

**DOI:** 10.3390/s19122733

**Published:** 2019-06-18

**Authors:** Arijit Ukil, Antonio J. Jara, Leandro Marin

**Affiliations:** 1 Research and Innovation, Tata Consultancy Services, Kolkata 700156, India; 2 Institute of Information Systems, University of Applied Sciences Western Switzerland (HES-SO), 3960 Sierre, Switzerland; 3 HOP Ubiquitous, 30562 Murcia, Spain; 4 Area of Applied Mathematics, Department of Engineering and Technology of Computers, Faculty of Computer Science, University of Murcia, Campus de Espinardo, 30100 Murcia, Spain; leandro@um.es

**Keywords:** IoT, cardiac heart monitoring, edge analytics, anomaly detection, differential privacy

## Abstract

Remote and automated healthcare management has shown the prospective to significantly impact the future of human prognosis rate. Internet of Things (IoT) enables the development and implementation ecosystem to cater the need of large number of relevant stakeholders. In this paper, we consider the cardiac health management system to demonstrate that data-driven techniques produce substantial performance merits in terms of clinical efficacy by employing robust machine learning methods with relevant and selected signal processing features. We consider phonocardiogram (PCG) or heart sound as the exemplary physiological signal. PCG carries substantial cardiac health signature to establish our claim of data-centric superior clinical utility. Our method demonstrates close to 85% accuracy on publicly available MIT-Physionet PCG datasets and outperform relevant state-of-the-art algorithm. Due to its simpler computational architecture of shallow classifier with just three features, the proposed analytics method is performed at edge gateway. However, it is to be noted that healthcare analytics deal with number of sensitive data and subsequent inferences, which need privacy protection. Additionally, the problem of healthcare data privacy prevention is addressed by de-risking of sensitive data management using differential privacy, such that controlled privacy protection on sensitive healthcare data can be enabled. When a user sets for privacy protection, appropriate privacy preservation is guaranteed for defense against privacy-breaching knowledge mining attacks. In this era of IoT and machine intelligence, this work is of practical importance, which enables on-demand automated screening of cardiac health under minimizing the privacy breaching risk.

## 1. Introduction

It is a well-known fact that deaths due to cardio-vascular diseases (CVD) are the biggest killer of human life. More than 31% of human life loss is due to cardiac-related diseases [1]. However, CVD is preventable when the early warning sign is captured before the disease has manifested internally. The development and deployment of the computational method for preventive, opportunistic, early-warning cardiac health management ensure better prognosis and probably lower the number of human life loss due to CVD. Subsequently privacy-preserved data management enables higher acceptability to the patient community and related stakeholders.

With the large-scale availability of wearable sensors and powerful smartphones, the realization of the automated cardiac health system in the mobile platform is the need of the hour. In fact, the Internet of Things (IoT) has an important role to play for the realization of affordable cardiac health management solution using artificial intelligence, machine learning and signal processing techniques. In this paper, the focus is on developing (under IoT infrastructure-based architecture) a data-driven computational model of detecting cardiac abnormality from heart sound or phonocardiogram or PCG signals, where PCG signals are collected from wearable sensors. In fact, capturing of PCG signals through smartphones has been initiated quite a few years back and paved ways for IoT based integration to realize the E-health ecosystem consisting of all the stakeholders like doctors, hospitals, medical caregivers, clinical researchers for immediate, timely, remote investigation and for prompt screening, treatment and diagnosis. IoT is used as the infrastructure to allow the computational model (clinical inferencing and privacy analytics) to be deployed on the edge devices (like smartphone) or cloud and for the deployment of the E-health system. 

We propose predictive modeling in the presence of cardiac abnormality from PCG data, which enables the subject to get immediate medical attention rather than when symptoms surface externally. However, the sensitive healthcare data is to be privacy protected and we need to safeguard against sensitive data breaching risk, which requires to be on-demand, based on user’s choice on privacy protection [2]. Privacy protection cannot be indiscriminant and in order to shield the possibility of data starvation of few of the stakeholders (we refer them as non-critical stakeholders that include social engineers, medical data researchers, statistical surveyors, etc.), novel data characteristics-based privacy protection is proposed. When data of a user S can be found out as ‘one in the crowd’, lighter obfuscation is incorporated, while if that is ‘unique in the crowd’, stronger protection is provided. The proposed scheme is an integrated approach of clinical utility and privacy protection that derives cardiac condition (equivalently a classification task) as well as ensures controlled privacy protection of patient’s sensitive healthcare information.

One of the salient aspects of the proposed scheme is its applicability in the context of edge analytics. In order to warrant the suitability of deployment of analytics solutions in edge devices, we need to satisfy two important criteria:

1. When inferential analytics or the training model generation is performed at the edge devices, the model construction need to be lightweight, typically by shallow networks with manageable dimension in the feature space. In fact, analytics on the source or at the edge is required particularly in the absence of private cloud infrastructure due to data privacy and security issues: In this paper, shallow network-based supervised learning with very limited number of feature dimension (precisely, three features) is performed, which invariably satisfies the computational requirement of trained model generation at the edge devices.
2. Healthcare data, being sensitive in nature, privacy protection needs to be carried out at the data origin: Our solution is privacy controlled. User or the data owner has the right to privacy preserve her healthcare data in a transparent manner. One of the main criteria of privacy protection for sensitive data is to ensure that utility is preserved. In our context, the utility is described as the amount of information available from the privacy-protected data. More distortion leads to higher protection with lesser utility of transformed data, whereas less distortion invites more privacy attacks. The proposed privacy protection method attempts to obfuscate the sensitive data to ensure adequate protection is made while utility is not severely compromised.

Our main intention is to construct an accurate model of clinical analytics over PCG signals, such that the inference it draws is capable to imbibe confidence to the patient as well as to other stakeholders like doctors, medical caregivers. On the other hand, the important features and inferences provided by the clinical analytics algorithm need to be privacy-protected while sharing with non-critical stakeholders who are not directly involved with the treatment or diagnosis specifically when the user or patient is conservative with respect to her privacy requirements. Our method not only constructs clinically reliable computational model, but also provides on-demand privacy protection as per user’s requirement. Thus, the proposed privacy protected integrated analytics method is positioned for practical acceptance to both patient and medical communities. The workflow of the approach is: 1. Capturing PCG signal locally or through Internet from PCG sensor, 2. Analyze and develop the clinical computational model at the edge or cloud from the training PCG signals, 3. Deploy the trained model at the edge or cloud, 4. Clinical analytics module provides inference as well as distinct features from field (or test) PCG signals, 5. User and other stakeholders like doctors, clinical researchers, hospitals access the outcome of the clinical analytics model pro-actively (by entering the analytics platform portal) or reactively (alarms sent by the platform to the critical stakeholders like doctors, hospitals) when inference is ‘Abnormal’, 6. User sets the privacy requirement. When privacy requirement is set ‘1’, obfuscation of the distinct features is made for the non-critical stakeholders like clinical researchers and inference is eliminated. Non-critical stakeholders only access the privacy-preserved features without any inference. 

Hence, we require 1. Powerful analytics method to ensure that cardiac condition is accurately inferred from the PCG signals so that alarm signals fetch immediate medical service for timely treatment, 2. Privacy-controlled information sharing with non-critical stakeholders to minimize the privacy-breaching risk of sensitive health information. 

This paper is organized as follows. In Section 2, related works and background material is presented, where we find that separate works on clinical analytics and data privacy protection are available with mature research outcome, an integrated approach, a critical requirement is yet not developed. The architecture of the proposed system is described in Section 3. In Section 4, our clinical analytics method is discussed which identifies clinical abnormal subject from PCG signal. In Section 5, novel privacy analytics algorithm is depicted that obfuscates the sensitive healthcare data when the subject demands. In Section 6, the efficacy of the proposed model is demonstrated through extensive experiments over expert-annotated, publicly available MIT-Physionet Challenge 2016 data [3]. Finally, the paper is concluded in Section 7.

## 2. Background

Our work has two main parts: 1. Analytics task: It provides classification of the physiological sensor signal (here, PCG) to detect the abnormal cardiac condition, 2. Privacy protection: It ensures that risk of sensitive data (e.g., features and inference from clinical analytics module) disclosure to unintended or non-critical stakeholders are minimized. These two parts are integrated. The analytics part enables clinical utility, while the privacy protection module warrants wide scale acceptance and practical importance. 

The proposed clinical analytics part attempts to detect anomalous cardiac condition or cardiac ailments by analyzing PCG signals. Automated analysis for cardiac disease identification research has been performed from the last few years. Good numbers of cardiac markers are used to unobtrusively assess the cardiac condition. PCG is one of the fundamental cardiac markers. PCG is a sound signal that captures the heart sound and murmurs. PCG signal carries significant information of cardiac activities. Cardio vascular diseases (CVD) change the state of the heart in terms of contractility and rhythm. PCG signal has different states: S1-Systole-S2-Diastole [3]. PCG signal from patients suffering from heart diseases carry additional heart sounds or murmurs. Earlier spectral estimation based techniques were used to analyze PCG signals [4], where authors have attempted to find additional heart sounds apart from regular S1, Systole, S2 and Diastole. Authors have exploited the spectral characteristics of PCG like strong frequency components, location of S1, S2 and temporal information on the S1–S2 interval are estimated to understand the presence of abnormal heart sound. With the rapid progress of machine learning paradigm, a good amount of research work is witnessed that demonstrates the effectivity of PCG signal for detecting cardiac abnormal subjects. For example, supervised learning or specifically, support vector machine (SVM)-based cardiac abnormality detection from PCG signals is presented in [5]. Authors in [6], extended SVM-based classification with modified cuckoo search and Linear Predictive Coding coefficients as features. Neural network based learner model construction with wavelet coefficients of PCG signals for cardiac condition identification is described in [7]. Sparse coding with dictionary matrix and corresponding sparse coefficient matrix feature-based SVM learning for PCG signal classification is reported in [8]. Fisher’s discriminant analysis from 90 different features is investigated in [9] for PCG signal analysis. In [10], authors attempted to extract 131 temporal, spectral, wavelet and statistical features from PCG signal and applied ensemble classifier to detect cardiac abnormality. We find [11] as the most relevant stat-of-the-art solution regarding the clinical analytics investigation. In [11], authors have proposed clinical analytics algorithm on PCG signals and experimented with same MIT_Physionet datasets [3]. Authors have first discovered important features among large pool of initial feature superset using minimum Redundancy Maximum Relevance (mRMR) algorithm [12]. The top five features from mRMR criteria are selected and fed to different classifiers like bagging- boosting based classifiers, SVM classifier with Radial Basis Function (RBF) kernel. The interesting part of the solution in [11] is the majority voting based decision among five classifiers. We have considered this algorithm as the state-of-the-art for comparative study of the clinical analytics outcome primarily due to its focus on PCG signals and experimentations on same datasets. 

It is understood that healthcare applications require strong privacy preservation [13]. Earlier, hospitals and clinics store and process entire healthcare records. Currently, the bulk of data is generated directly from the patient side and often the hospital is simply acting as the record containing entity [14]. This patient-centric shift of healthcare data processing needs for a patient-centric privacy preservation [14]. With the advent of IoT-based patient care, local privacy protection becomes the need of the hour [15,16]. The paradigm shift of centralized privacy protection to distribute and local privacy protection necessitates different approach of patient-data privacy mechanisms. For example, locally generated healthcare data (say, PCG signal or feature sets) may be differently privacy-preserved based on the destination profile. When destination of the information is registered, the doctor or hospital privacy protected outcome is different than when the destination is clinical data research wing of a public organization or medical science research fellows. In order to introduce controlled data privacy such that the social science aspect of it (where users or patients or subjects voluntarily participate in the clinical survey) is not compromised, we find that differential privacy is the right tool. Differential privacy does not increase the risk while participating in statistical database [17]. We are further motivated by the investigation of [18], which observes that on-demand, differential privacy protection of healthcare data and integrated approach of privacy protected clinical analytics are critical requirements to build IoT-enabled automated health screening system. It is witnessed that such a requirement is yet to be captured and a suitable solution is not proposed. The adoption of IoT for the development of E-health systems is a necessity to achieve increased ambulant and remote medical care, reduced cost, increased quality and better personal care [19]. It is indeed recognized that remote cardiac care and early screening result in better prognosis of cardiac related ailments and IoT seems to be the appropriate platform to enable the remote heath care services. When private and secure channel of data communication is enabled [20], E-health eco-system becomes more acceptable to all the stakeholders particularly to the patient community. 

One of the main contributions of this research work is to develop an on-demand privacy protection scheme such that information content of shared sensitive data is a function of the profile of the destination. For instance, when the profile of the destination is a registered doctor, complete data is shared and when the destination is a clinical researcher (who does not get involved in patient’s healthcare, but needs data for her research work and statistical surveying, statistical correlation establishment), properly obfuscated data is shared. However, it is to be kept in mind that anonymization [21] reveals sensitive data under homogeneity and background knowledge attacks [22]. Our method does not rely on simple anonymization-based privacy protection, we propose differential obfuscation-based privacy protection to develop a data-driven clinical analytics solution on healthcare data and to demonstrate the efficacy of an integrated scheme. Our privacy definition is that adversary’s learning from the user’s ’private’ data (like the inference from the computational model, distinct features), does not substantially increase with additional set of data, i.e., prior and posterior probability of finding private information does not change beyond a threshold ϵ, which is called the privacy factor. We achieve this privacy guarantee through differential privacy [17,23]. In fact, differential privacy is emerged as the appropriate model to protect healthcare data [18].

In order to deploy the proposed architecture, IoT provides the most suitable platform. Sensor data is fed to the edge devices like smartphone, where clinical analytics and privacy protection mechanisms are deployed. The edge device also acts as the Internet gateway. Other stakeholders access the privacy-protected information through a dedicated portal or mobile application. Based on stakeholders’ credential, privacy protection is enabled. Users set the privacy requirement and accordingly, data obfuscation based privacy protection is enabled. Data transit is made through a secure channel with secure transport protocol like “https”. 

## 3. Architecture

**Figure 1:**
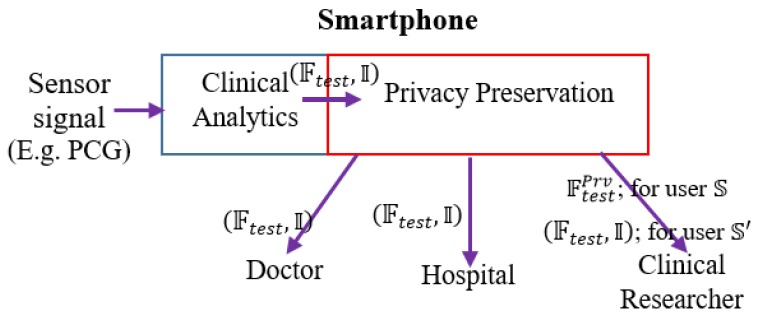
Data-driven on-demand privacy preserved clinical analytics: Functional architecture.

We envision the following functional architecture for data-driven, on-demand privacy preservation of healthcare data (Figure 1). It is assumed that the analytical investigation of clinical data, captured from sensor device is performed at the edge, typically in a smartphone. Smartphone employs powerful data-driven algorithms for providing basic clinical analytics outcome. The main idea is the privacy preservation of the clinical analytics outcome based on the user’s preference. First, the trained model is developed with the given PCG signals. The constructed trained model is deployed. Test PCG signals are fed to the trained model. Let, the outcome over the tests signals be in the form of features on the test or field data and inferences: $O=\left(F_{test},\;I\right)$. For example, features F may be the distinct properties of the data or analytics solution, which could be mean heart rate, heart rate variability and others of clinical significance, inference (decision like whether the PCG signal is from cardiac ‘normal’ or ‘abnormal’ subject). I could be a flag indicating whether the PCG signal is from cardiac healthy or abnormal person. Next is the privacy preservation module, which is based on the demand (or setting) from the user, enables required privacy protection. Privacy preservation transforms the clinical analytics outcome $O=\left(F_{test},\;I\right)$. S is a conservative user, she does not intended to disclose her clinical outcome $O=\left(F_{test},\;I\right)$ to the clinical researcher in an anonymized but raw form. For example, a subject S shares her PCG data to a number of stakeholders that include doctors, hospital, emergency service providers as well as the data management ecosystem include clinical researchers. However, S feels uncomfortable to share data with others except the medical caregivers and the hospital. In such scenario, S healthcare data is to be privacy preserved. In another case, S′ does not feel data privacy requirement and she allows her healthcare data to be shared to all the stakeholders. Privacy preservation module obfuscates $\left(F_{test},\;I\right)\to \left(F_{test}^{Prv}\right)$ and shares $F_{test}^{Prv}$ for user S. It is to be noticed that inference details are not shared when privacy preservation is required. In large number of scenarios, inference is binary, say pathological normal or abnormal. Hence we avoid sending binary information as obfuscation of such information is meaningless and consequently privacy preserved feature information ($F_{test}^{Prv})$ is shared when privacy setting is ON for the destination (here, non-critical stakeholders in healthcare ecosystems like clinical researcher) and inference information is omitted. 

Another user $S^{\prime }$ does not consider privacy on her data and perhaps, intends to enhance the knowledge of clinical researchers. In such scenario, $\left(F_{test},\;I\right)$ is shared to the clinical researchers for user $S^{\prime }$. Based on the privacy setting P of the user, obfuscation is used. However, privacy preservation is not performed for critical stakeholders like medical caregivers, doctors and assigned persons from designated hospitals for any type of the users.

Our architecture provides complete control of data privacy on the hand of the patient or originator of the data (whose privacy is the real concern). Such kind of de-risking system surely induces much more acceptability to the remote health screening and medical service provider eco-system. Another important feature of our system is the complete transparency of the private data flow. When privacy is intended, private data flow is restricted to the assigned (=private) stakeholders like doctors or nurses. Contrary situation is when the user intends to participate in submitting clinical data and inference to the clinical research community (=public domain), private data is not obfuscated. One of the pertinent questions arise: $F_{test}^{Prv}$ could have been garbage data consists of randomly generated sample points with dimension $\left|F_{test}^{Prv}\right|$. However, we assume that clinical researchers have a certain idea on the distribution of $F_{test}^{Prv}$ and considering clinical researchers may contain potential privacy attackers, $F_{test}^{Prv}$ is to be obfuscated such that distribution preservation is maintained while injecting noise. Thus, our architecture caters the need of remote health management under patient-controlled privacy setting with a high degree of private data breach de-risking.

We depict this scenario of on-demand privacy preservation of clinical analytics in Figure 1. From the PCG signals, the clinical analytics (from its trained model) infers or provides decision (say, whether the sensor signal belongs to normal or abnormal subjects) I along with extracted test features $F_{test}.\;$ Based on the user’s privacy preservation preference, $F_{test}$ is transformed to $F_{test}^{Prv}$. When the user is conservative (user S), only $F_{test}^{Prv}$ is shared to clinical researchers or non-critical stakeholders, for the non-conservative user (user S′) transformation is not performed $\left(F_{test},\;I\right)$ is shared.

**Figure 2:**
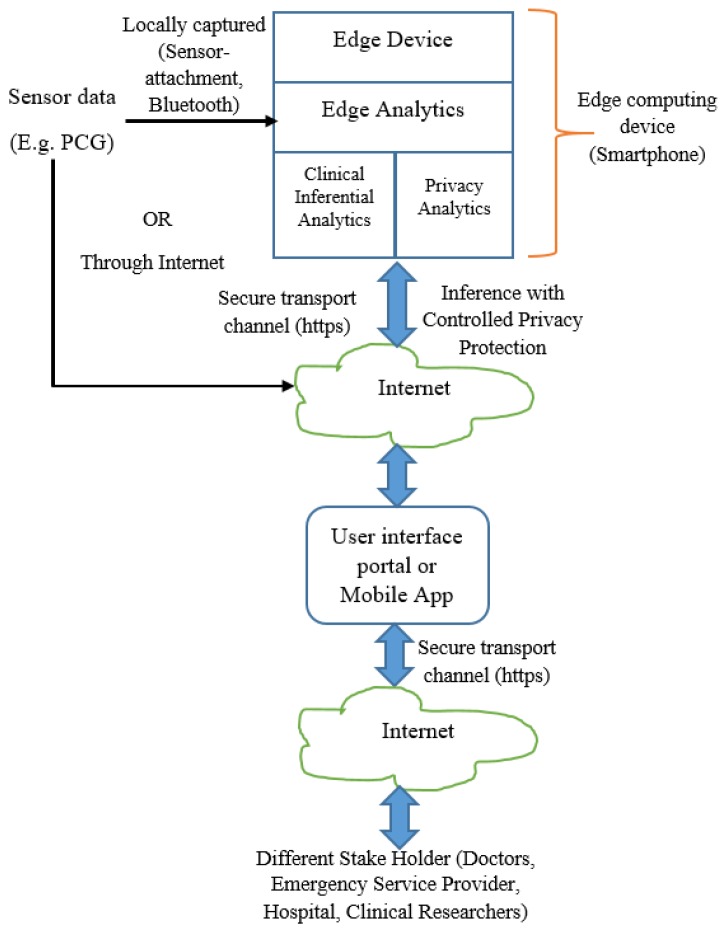
Smart edge analytics for clinical inference and controlled privacy protection: Deployment architecture.

In the context of edge analytics, we depict the deployment architecture as per Figure 2. The inferential model and controlled privacy protection are performed at the edge devices. The stakeholders including the data owner receive the inference and related information from the edge devices through Internet. The data owner may receive the information locally (when analytics is performed in her smartphone or other edge computing devices). 

Our subsequent discussion is based on the functional architecture (Figure 1) and deployment architecture (Figure 2). When sensor data is fed to the analytics engine, the computational model consisting of feature set and trained model are generated. The generated model is deployed for prediction. It is envisaged that the trained model generation and trained model deployment are both developed and deployed on edge devices like smartphone. One of the salient aspects of our architecture is its capability to provide substantial clinical utility along with protecting the privacy requirement of the user. It is indeed an integrated method to ensure privacy protected clinical utility. Data at transit is protected through secure transport layer protocol (https). PCG signal captured by the sensors is sent locally or through the Internet. All the stakeholders access the information through a dedicated portal or mobile application. 

## 4. Clinical Analytics

We employ a machine learning-based approach for clinical analytics. It involves two major steps: 1. Feature space extraction, and 2. Learning using classifier model. Firstly, the trained classifier is constructed from the training data set and generated learning model is used to detect whether the test PCG signal is from clinically normal or abnormal subjects. The proposed clinical analytics module identifies pathological abnormality from the sensor captured PCG signal and shares the inference (I) along with extracted features to the privacy preservation module, which according to the privacy setting of the user, share information O to the stakeholders. 

Let, $X\;\subseteq \;R^{n}$ be *n*-dimensional instance space, feature space F, target class or label space L= {±1}, where +1 indicates clinically normal and -1 indicates clinically abnormal; prediction space $\hat{L}=\;\left\{\pm 1\right\}$, training example space $T=\;\left\{\left(x_{1},\;l_{1}\right),\left(x_{2},\;l_{2}\right),\;\ldots \;\left(x_{n},\;l_{n}\right)\right\}\;\in \;{\left(X\times \left\{+1,\;-1\right\}\right)}^{n}$, where n = total number of training examples. Let, ${𝒻}_{1},\;{𝒻}_{2},\ldots ,\;,\;{𝒻}_{\Upsilon }$ form the feature space F of Υ number of features.

In this work, the ensemble learning classifier Adaboost is considered [24]. Ensemble learners consist of a number of weak hypotheses ${𝓀}_{i},\;i=1,2,\ldots ,\;H$ and form an ensemble hypothesis $E\left({𝒻}_{k}\right)$, for the k^th^ feature ${𝒻}_{k}\in F,$ where

(1) $$E\left({𝒻}_{k}\right)=\;\overset{H}{\underset{i=1}{\sum }}{𝓀}_{i}\left({𝒻}_{k}\right)\times \varrho_{i},\;\forall {𝒻}_{k}\in F$$

$\varrho_{i}$ are the weights of each of the constituent learners.

Adaboost is an iterative process and it minimizes the error Δ(ϱ) at each of the iterations [21].

(2) $$\Delta \left(\varrho \right)=\;\sum_{k}\sum_{i}e^{\left(\left({𝓀}_{i}\left({𝒻}_{k}\right)\times \varrho_{i}\right)+\varepsilon_{i-1}\;\left({𝒻}_{k}\right)\right)},\;\forall {𝒻}_{k}\in F$$

(3) $$\varepsilon_{i-1}\;\left({𝒻}_{k}\right)=\;\sum_{i=1}^{H-1}{𝓀}_{i}\left({𝒻}_{k}\right)\times \varrho_{i},\;\forall {𝒻}_{k}\in F$$

**Figure 3:**
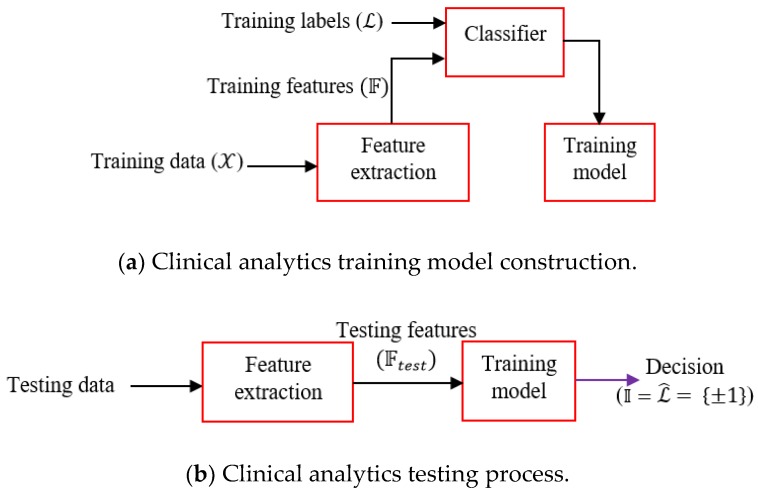
Clinical analytics approach.

We choose boosting learners as it is often found that boosting does not suffer from an overfitting problem particularly due to the presence of number of weak learners. The learning workflow is depicted in Figure 3. First, the training model is constructed from the training features and associated labels. The trained model is deployed for field-testing, where the features of the test signals are fed and the model provides the decision. Clinical inferential analytics: Firstly, the features F are extracted from the training data X. Training features and corresponding training labels L are fed to the classifier (Adaboost) and the trained model is generated. Testing is done by first extracting the features from test data and extracted test features $F_{test}\;$are fed to the trained model. The trained model predicts decision $\hat{L}$, equivalently inference I, where $I=\hat{L}.$

The above scheme (Figure 3) is a generic representation of our clinical inferential analytics. Let, training PCG data set be {X} and number of features from X is derived. The feature space F consists of three features: $F=\left\{{𝒻}_{1},\;{𝒻}_{2},\;{𝒻}_{3}\right\}$. In this particular case, we consider Υ = 3, where ${𝒻}_{1}\;$is kurtosis $\left(x_{i}{}^{2}\right)$, ${𝒻}_{2}$ is max(absolute(Hilbert Transform $\left(x_{i}\right))$, ${𝒻}_{3}$ is max(mean(power spectra($x_{i}$))), ∀i∈n. It is to be noted that feature space transformation maps the signal to a vector of dimension 3. Let, the training signal space is $\left[x_{1},x_{2},\ldots ,x_{n}\right]$, where $x_{i}\in R^{d},\;i=\left[1,2,\ldots ,n\right].\;$Feature space maps the signal space $R^{d}\to R^{\Upsilon }$, Υ is the dimension of the feature vector, currently, Υ=3. The feature set is described below.

- ${𝒻}_{1}:\;$ Kurtosis (data^2): $Ε\left[\frac{{\left(x_{i}{}^{2}-\mu_{i}\right)}^{4}}{\sigma_{i}{}^{4}}-3\right]$, ∀i∈n
  Intuition: Kurtosis is a standard statistical measure of the “peakedness” of the probability distribution. When the underlying distribution of $x_{i}$ kurtosis value is more (>3), which indicates leptokurtic property, the Squared sample points of the signal space ($x_{i}{}^{2}$) amplify the outliers and kurtosis statistics reveals the outliers more prominently.
- ${𝒻}_{2}:\;$ Max(abs(Hilbert(data))): Max(abs(Fast Fourier Transform($x_{i}))),\;\forall i\in n$.
  Intuition: Using Hilbert function, the exact analytic signal for $x_{i}$ is computed. Hilbert transform of $x_{i}\;$computes the instantaneous attributes of the amplitude and frequency of signals [25]. The maximum value of the analytic signal finds the highest contributor of the signal disturbance.
- ${𝒻}_{3}:\;$ Max (mean(power spectrum(data)): Max (mean($S_{xx}\left(fr\right)$), where $S_{xx}\left(fr\right)$ is the power spectrum with frequency variable fr which is calculated from each $x_{i}$ as the Discrete Time Fourier Transform of the covariance of the slided windowed segment of $x_{i}$ [26].
  Intuition: Power spectrum of estimates over the defined number of sliding windows to understand the spectral (frequency components) change over time. PCG is a non-stationary signal. $S_{\mathrm{xx}}\left(fr\right)$ estimates the short term periodogram, which discloses the degree of non-stationarity in PCG signals. It is assumed that $S_{\mathrm{xx}}\left(fr\right)$ estimation of anomalous ECG signals is more than regular PCG signals.

In the case of Adaboost algorithm, we need not provide any threshold for classification; based on the training data distribution Adaboost forms the hypothesis or trained model. The constructed trained model is used for testing purposes.

**Figure 4:**
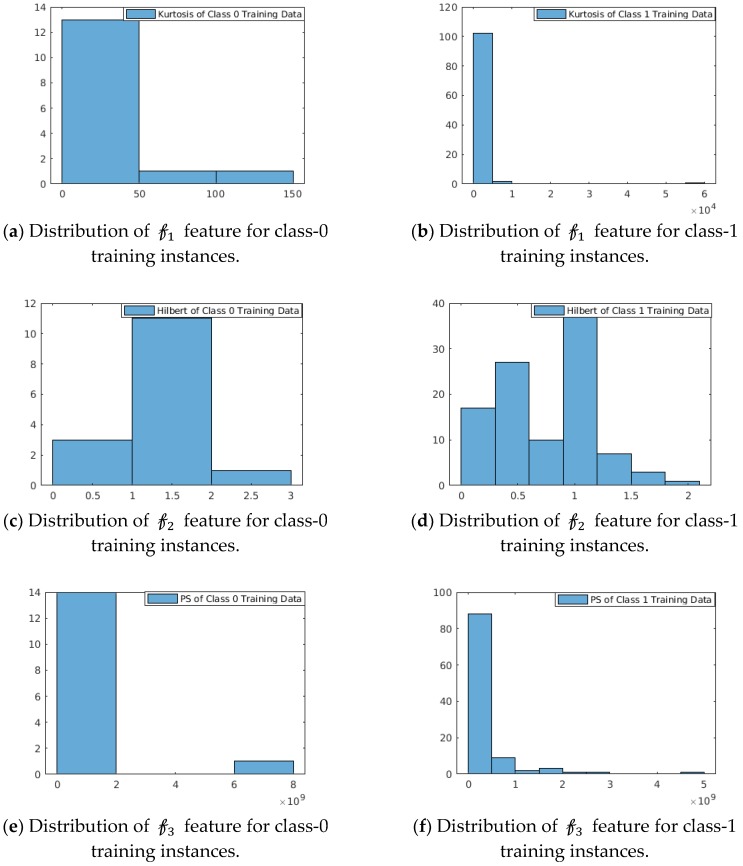
Strength of the three features ${𝒻}_{1},\;{𝒻}_{2},\;{𝒻}_{3}\;$respectively are shown. The probability distribution over the training datasets of the selected features for two different classes demonstrate distinctive characteristics.

Strength: The strength of these features is shown in Figure 4. We have considered PCG datasets from the MIT-Physionet Challenge 2016 [3]. The probability distribution of this feature space as depicted in Figure 4 shows that the features are capable to distinguish the two classes. We observe:

- For ${𝒻}_{1}:\;$ In the feature function kurtosis (data^2), class-0 training instances are concentrated over (0, 50) with a right-sided tail spreading with range (0, 150) of feature values, whereas for class-1 training instances are concentrated in narrow-band over (0, $\;6\times 10^{4}$) with insignificant right-sided tail.
- For ${𝒻}_{2}:$ The feature function Max(abs(Hilbert(data))) shows multi-modal distribution for class-1 training instances, whereas class-0 training instances show approximately unimodal distribution with mean at 1.5.
- For ${𝒻}_{3}:$ In the feature function Max (mean(power spectrum(data)), class-0 instances are concentrated in (0, $2\times 10^{9}$), whereas class-1 instances are distributed over the range (0, $5\times 10^{9}$).

## 5. Privacy Analytics and Sensitive Data Obfuscation

Our endeavor is to address the health data privacy preservation issues in a holistic manner. We assume that a proper secure channel like the secure transport protocol (https) or other kind of security mechanism [27] is established when data is in transit (as depicted in Figure 2). The main objective is that user’s privacy should be preserved if the user wants privacy protection when sharing sensor data (here, healthcare data example is PCG signal features) such that sensitive data is obfuscated to warrant sufficient information loss to the attacker. However, healthcare data has an extensive role in the scientific community, specifically for medical science research. In such scenario, our attempt is to obfuscate sensitive data when it is deemed necessary and demanded by the user. Our novelty of the approach is that we devise an intuition that when sensitive information is ‘one in the crowd’, obfuscation may not be required or weak obfuscation is sufficient since sharing that information does not enrich the attacker’s available knowledge. Whereas, when something or more precisely the sensitive data is ‘unique in the crowd’, that sensitive information needs strong obfuscation as that the available information enhances the knowledge of the attacker. For example, the attacker knows that Mr. X is admitted in a hospital that specializes in cardiac care. Here the knowledge from the ‘crowd’ is that Mr. X suffers from cardiac diseases. If Mr. X suffers from cardiac disease and that information is retrieved by the attackers, attacker’s knowledge gain →0. However, if Mr. X suffers from cancer and is admitted to the cardiac care specialty hospital that information is unique and should be appropriately obfuscated. Thus, we ensure that the information sharing is not completely compromised and some of the stakeholders (most likely the medical research community) do not starve. However, the caveat is that the knowledge mining capability of certain stakeholders are minimized on patient’s demand for privacy protection.

In order to identify whether the information (here $F_{test}$) is unique in the crowd, we first identify the anomalous behavior probability of the feature among the already available set. We employ the Density-based spatial clustering of applications with noise (DBSCAN) algorithm [28] to mine the distribution of data under investigation. DBSCAN is a density-based clustering non-parametric algorithm that finds the outliers. Let, Ω be the data points (in our case it is the features from the test sample, $F_{test}$), which is data under investigation and it is a result of feature transformation function: J → Ω $\left(=F_{test}\right)$. In the DBSCAN algorithm, two parameters ϖ,𝓃 are to be tuned, where ϖ,𝓃 are the distance and density parameters respectively, where ϖ is defined the furthest distance for which a point is density-reachable and 𝓃 is the minimum number of points required to form a density cluster [28]. We choose 𝓃 as proposed in [28], i.e., 𝓃=4, irrespective of the distribution of Ω. It was shown in [28] that for k>4, the k-dist graphs do not considerably differ from the 4-dist graphs. We set, 𝓃=4 as it is sufficient for accurate analysis [28]. Our heuristics of finding ϖ differs from [28] as follows. We propose that ϖ= 3σ, σ = standard deviation (Ω). The underlying philosophy is: Let D be the distance of an arbitrary point ρ in Ω to its at least 𝓃−1 nearest neighbors, so the D-neighborhood (spherical) contains at least 𝓃 points. The most probable value of (even if Ω≠N(μ, σ)) ϖ (=D) for least false positive is ϖ= 3σ. Let $\Omega^{1},\;\ldots ,{\;\Omega }^{K}$ be the K clusters following ϖ, η. Anomalous sensor data points Ω′ are the set of sensor data points in Ω that are not part of any $\Omega^{k}$, k= 1,2, …. K.

Our main contribution is to effectively delineate and implement the sensor data uncertainty principle. We define the sensor data uncertainty principle as to introduce (statistical/ information theoretic) uncertainty of sensor data when analytics is used for summarization, performance prediction, inference, trend estimation by the attacker or adversary. Our privacy protection definition is that adversary’s learning from the user’s sensor data does not substantially increase with additional set of data, i.e. prior and posterior probability of finding private information does not change beyond a threshold ϵ. 

Let $\Theta =\left\{\theta_{i}\right\}$, i = 1,2,3… be the set of functions on Ω. Assuming Ω be finite, $\Omega \overset{PPDM}{\to }\;\Omega^{P}$, such that there exists no computationally feasible function or mining technique in Ω that guarantees $\Omega^{P}\to \;\Omega \;$without uncertainty probability ϵ. (PPDM is abbreviated from ‘Privacy Preserving Data Mining’).

This privacy criteria is achieved through differential privacy [17]. The testing or field features $F_{test}$ are to be privacy-preserved. Hence, $F_{test}$ are made ϵ-differentially private $\Omega^{P}\left(=F_{test}^{Prv}\right)$, where ϵ is called the privacy factor.

(4) $$\Omega \left(\Omega =F_{test}\right)\overset{\Delta PPDM}{\to }\Omega^{P}\left(=F_{test}^{Prv}\right)$$

where, ΔPPDM is the differential PPDM of Ω.

We define differential privacy on Ω to form ϵ-differentially private $\Omega^{P}$ as [17] for arbitrary computational function f and S ⊆Range (f).

(5) $$\frac{Pr\left[f\left(\Omega \right)\;\in \;S\right]}{Pr\left[f\left(\Omega^{P}\right)\;\in \;S\right]}\leq \;e^{\epsilon }$$

In order to introduce differential privacy, appropriate perturbation or noise addition on Ω to form $\Omega^{P}\;$is required and the Laplacian noise is chosen as the perturbation method.

Laplace noise is defined as: $lap\;\left(\Omega \right)=\;e^{\left(\frac{\left(\frac{-\left|\Omega \right|}{b}\right)}{2b}\right)}$, where, standard deviation (Ω) = $\sqrt{2\;}\;b,\;$in laplacian noise addition, noise distribution depends on ΔΩ and ϵ. However, it is independent on the distribution of Ω. In order to achieve more distortion on $\Psi \left(\Omega^{P}\right)$, sensitivity parameter ΔΩ should be higher. Our intention is to achieve symmetric noise for achieving ϵ− differential privacy, we use lap (b), where $b=\;\frac{\Delta \Omega }{\epsilon },\;\Delta \Omega =\;\underset{adj\;\Omega ,{\;\Omega }^{P}}{\max}\left|f\left(\Omega^{P}\right)-f\left(\Omega \right)\;\right|.$ It is proved that adding laplacian noise with $b=\;\frac{\Delta \Omega }{\epsilon }$ ensures ϵ−differential privacy.

Let, J  be the differential private information. According to the definition of ϵ−differential privacy [17] we need to find whether $\frac{Pr\left[\left(f\left(\Omega \right)+lap\left(b=\frac{\Delta \Omega }{\epsilon }\right)\right)=J\right]}{Pr\left[\left(f\left(\Omega^{P}\right)\;+lap\left(b=\frac{\Delta \Omega }{\epsilon }\right)\right)=\;J\;\right]}\leq \;e^{\epsilon }.$

We choose ϵ=3σ, σ = $\mathrm{standard}\;\mathrm{deviation}\;\left(\Omega \right),\;\Omega =F_{\mathrm{test}}.$ The intuition is that privacy preserved transformation sufficiently but not drastically obfuscate the sensitive data Ω to construct $\Omega^{P}$. When ϵ is high, obfuscation leads to random outcome, which is not intended or when ϵ is low, too much exposure of Ω in $\Omega^{P}.$ The idea of distortion based on the principle of ‘unique in the crowd’ is to find the outliers by the DBSCAN algorithm [1] for distance variability ϖ= 3σ, σ = standard deviation (Ω). Consequently, privacy factor ϵ=3σ ensures sufficient but not extreme distortion on the distribution of Ω. Thus, utility of the information is not reduced substantially while privacy protection is achieved. 

**Figure 5:**
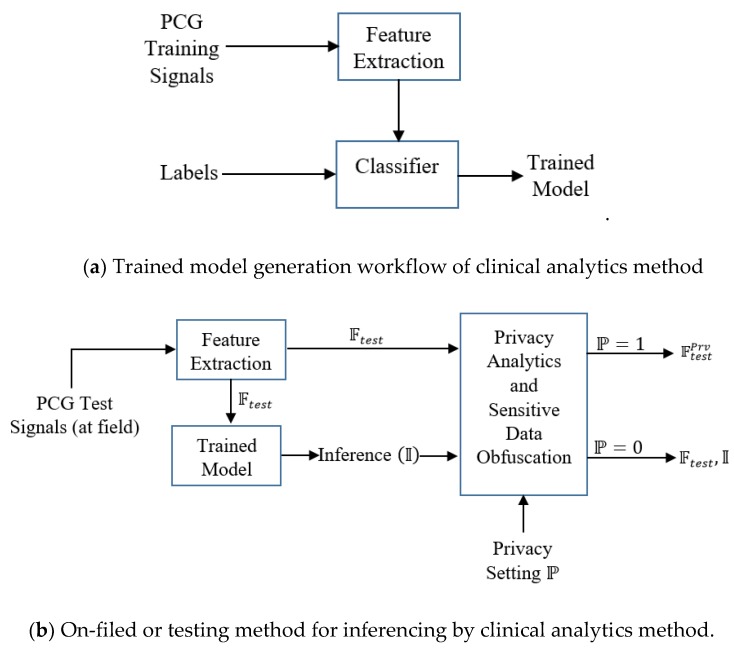
Functional workflow of the proposed method.

Let, P be the privacy setting for the user. When the user sets P=1 for certain stakeholder, privacy preservation is performed on the significant feature set Ω $\left(=F_{test}\right)$ and $\Omega^{P}\left(=F_{test}^{Prv}\right)$ is shared to that stakeholder. Others receive Ω $\left(=F_{test}\right).$ When P=0, the derived feature set F as well as the derived inference I are shared with all. We like to emphasize that the proposed controlled private data obfuscation results in disturbing the critical information (which is the outliers in the signal that act as the marker of indicating the presence of disease). Such controlled obfuscation results in minimum disturbance to the distribution of the signal characteristics. Hence, $F_{test}^{Prv}$ masks the critical information without affecting the overall distribution. The workflow description of the proposed method is depicted below in Figure 5. The steps are:

- Features ($\left\{{𝒻}_{1},\;{𝒻}_{2},\;{𝒻}_{3}\right\}$) are extracted from the available PCG training signals.
- Training features along with the corresponding labels (L) are fed to the classifier (Adaboost) with number of trees equal to 100.
- Trained model is generated.
- Test PCG signals or on field PCG signals are fed to the feature extraction module. Testing features $F_{test}$ are generated and fed to the trained model.
- Inference (‘normal ‘ or ‘abnormal’) is generated and fed to the Privacy Analytics and Sensitive Data Obfuscation module. Based on the privacy setting P  (when P=1) of the user, either the obfuscated feature set $F_{test}^{Prv}$ using differential privacy without inference or the derived raw feature set $F_{test}$ along with the inference decision I is sent to the non-critical stakeholders. Critical stakeholders like doctors and medical caregivers are always provided with $F_{test}$ and I irrespective of the privacy setting.

## 6. Results and Analysis

Our main focus of this work is to demonstrate that:

1. Clinical efficacy is significant when computational methods are employed through data-driven techniques. We have considered heart sound or PCG signal as the healthcare data source and detection of cardiac abnormality from the PCG signal is the inference outcome along with few important cardiac markers.
2. Based on the user’s demand, privacy preservation is employed on the feature set with the constraint that arbitrary obfuscation on sensitive data is to be minimized.

**Figure 6:**
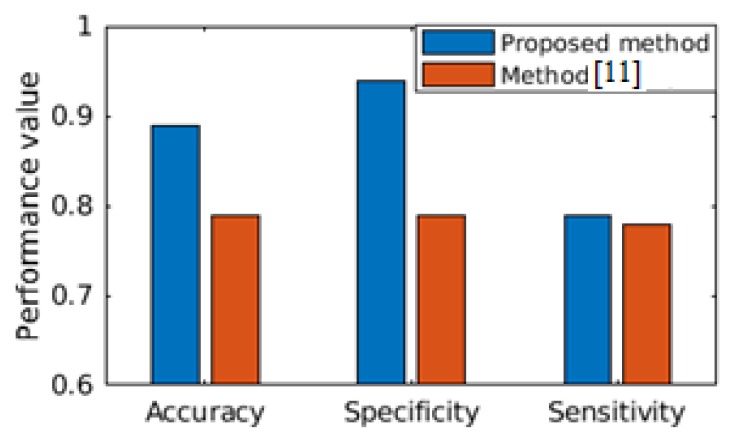
Performance comparison of proposed clinical analytics method of detecting clinical ‘abnormality’ from PCG signals.

**Figure 7:**
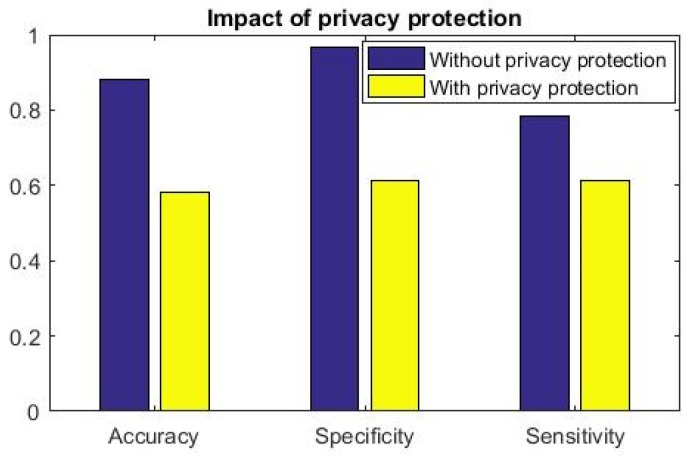
Clinical analytics results of the proposed data-driven method and impact of privacy preservation. It shows that the proposed inferential analytics method (result of without privacy protection when shared data is $F_{test}\;$) produces significant clinical efficacy (inference I): Accuracy, sensitivity, specificity all more than 0.8. Our proposed privacy preservation technique obfuscates the features such that clinical efficacy (inference $I^{Prv})\;$ on obfuscated data $F_{test}^{Prv}$ drops significantly (almost random outcome, performance of merit →0.5 when inference decision is made while protecting data privacy.

**Figure 8:**
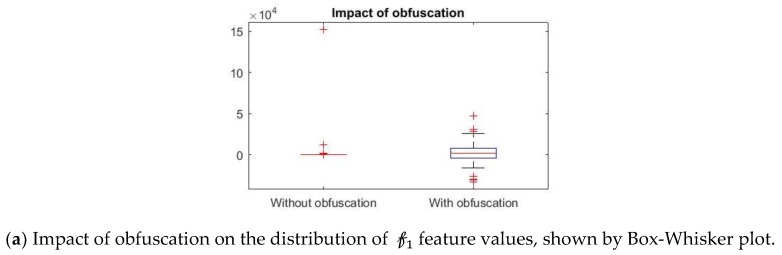

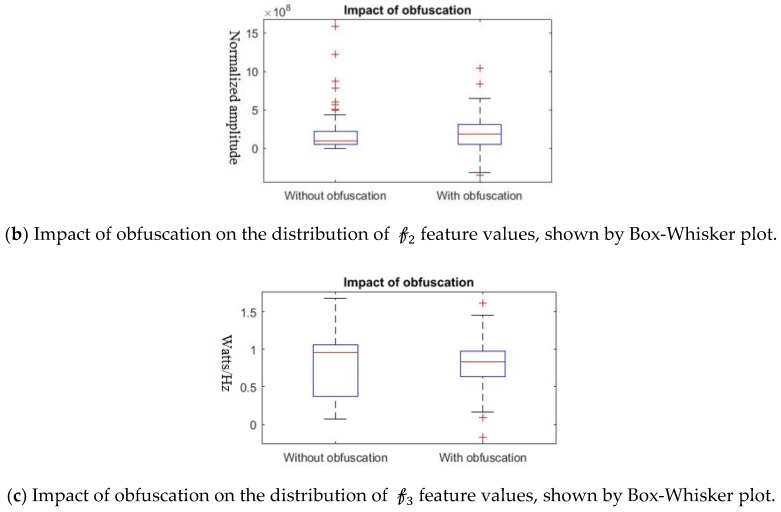
Statistical significance and impact of obfuscation of three features ($\left\{{𝒻}_{1},\;{𝒻}_{2},\;{𝒻}_{3}\right\}$) in $F_{test}\;$ and $F_{test}^{Prv}\;$ respectively.

**Figure 9:**
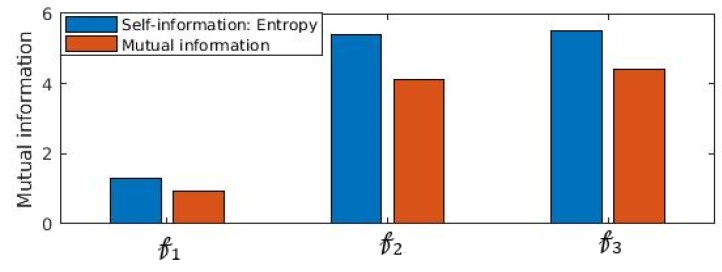
Impact of obfuscation in mutual information for three features $F_{test}\;$respectively. We observe that $𝒻\left(F_{test};\;F_{test}^{Prv}\right)\;<I\left(F_{test};\;F_{test}\right)$: Mutual information between the original feature $F_{test}$ and privacy preserved feature $F_{test}^{Prv}$ is less than the entropy or self-information between the raw features.

Let, $F_{test}\;$and $F_{test}^{Prv}$ are shared to the attacker A and A intends to derive inference from the available feature set. It is assumed that A possesses the data-driven clinical analytics tool as described in Section 4. We present the results to highlight that the proposed clinical analytics method is powerful enough for accurate detection of cardiac abnormality from PCG signals. We show the performance efficacy of the proposed model in Figure 6. Hence, the clinical analytics method provides reliable inference as part of the clinical inference analytics of edge analytics systems (please refer Figure 2). We assume that the attacker A also uses this powerful analytics method to infer the cardiac condition from PCG signals. Health condition information being sensitive in nature, the privacy revealing knowledge $F_{test}$ needs to be protected when shared to non-critical stakeholders like clinical researchers. The proposed differential-privacy based obfuscation of $F_{test}\;$and in case the user does not intend to share privacy revealing knowledge $F_{test}$ to non-critical stakeholders, $F_{test}^{Prv}$ is shared. In Figure 7, Figure 8 and Figure 9, we show the efficacy of the proposed privacy preservation techniques. In Figure 7, we demonstrate that the inferencing capability of the analytics method is significantly reduced on $F_{test}^{Prv}.\;$ In Figure 8 and Figure 9, we analyze the causality behind the reduction of the analytics method.

We conduct our experimentations using publicly available, expert-annotated MIT-Physionet Challenge 2016 PCG large database [3]. MIT-Physionet Challenge 2016 PCG contains more than 3000 PCG signals with ‘Normal’ and ‘abnormal’ labels corresponding to clinical normal of cardiac condition and clinical abnormal of cardiac condition, respectively. Precisely, the dataset consists of 3126 heart sound recordings or PCG signals, lasting from 5 s to around 120 s with 2000 Hz sampling rate. The abnormal PCG datasets are mostly from patients suffering with heart valve defects and coronary artery diseases. The first part of our work is to demonstrate the clinical efficacy of the proposed analytics method, where the task is to classify the given PCG signal as ‘Normal’ or ‘Abnormal’. We use stratified K-fold cross-validation (K=5) for performance result demonstration. We further show comparative performance with relevant state-of-the-art solution [11]. In Figure 6, performance comparison between the state-of-the-art [11] is made, where both of the studies have considered the MIT-Physionet Challenge 2016 PCG large database [3] for demonstrating the capability of the algorithms. We find that our proposed method outperforms the state-of-the-art method in all the three performance metrics, viz. accuracy, specificity and sensitivity. Hence, we can safely assume that the proposed clinical analytics method for detecting abnormal cardio-vascular condition from PCG signal has a large potential to provide clinical analytics outcome. 

The second part of our work is to demonstrate that on-demand, effective privacy protection can be applied on the feature sets such that revealing of the privacy-preserved features do not provide the attacker to guess the clinical condition of the user or the subject. When an attacker A gets complete information F, A can derive the clinical inference I quite effectively with (For example, accuracy, sensitivity, specificity scores >0.8). We can surely claim that the inference engine has the capability of producing good learned model. Hence, we find a significantly accurate clinical inference. Such high accuracy inference ensures the minimization of both false negative and false positive alarm rates. The alert generated to the medical service provider is more reliable and subsequently the appropriate action ensures better prognostic. It is observed in Figure 6 that the proposed clinical analytics method is outperforming the relevant state of the art method [11] in all of the performance metrics like accuracy, sensitivity, and specificity. 

However, when information is obfuscated and attacker A receives $F_{test}^{Prv}$, inference effectivity drops substantially. More importantly, we observe that inference drawn from $F_{test}^{Prv}\;$(privacy protected features) has random outcome: Accuracy, sensitivity and specificity measures are →0.5 (Figure 7). This result further shows the efficacy of the proposed data-driven cardiac disease clinical analysis. When this analytics engine is part of the smartphone and inference is available locally to the patient, she can get information regarding her cardiac health and when found abnormal, she can immediately get treated by medical practitioners. This inference further alerts the registered doctors, emergency service provider to attend the patient when clinical inference I is shared real-time using IoT setup, whereas $I^{Prv}$ be the inference extracted from $F_{test}^{Prv}$. It is to be noted that clinical analytics performance of our learning method (i.e., inference I) is significantly accurate to ensure basic remote screening of cardiac health. 

We envisage that such ecosystem potentially creates a proactive privacy-enabled cardiac health management. Consequently, our architecture will pivot the path towards derisked remote health management. The generic construction of the learning method (the clinical analytics part) requires application specific feature selection. Otherwise, our proposed architecture is generic enough to be deployed as a part of remote health management eco-system. In fact, the privacy-preserved and privacy-controlled architectures enable the standard acceptance when practical deployment is to be implemented. It is noted that in Figure 7, the clinical analytics (although it is a powerful learned model) fails to properly infer when privacy preservation is performed, i.e., the trained model is fed with $F_{test}^{Prv}$.

Further, we show that the proposed differential privacy protection that obfuscates $F_{test}\;$and constructs $F_{test}^{Prv}$ that distorts the statistical properties of the data as shown in Figure 8. We use Box–Whisker plot to show that the distribution of data changes drastically with our obfuscation method, which confuses the attacker to derive effective inference. Box–Whisker plots show that the privacy-preserved feature space is transformed to a close to random outcome as well as significant statistical distortion. 

Another important statistical property that establishes statistical significance between distributions is mutual information. We show in Figure 9 that mutual information $I\left(F_{test};F_{test}^{Prv}\right)=\;\underset{F_{test,\;\;}F_{test}^{Prv}{}^{\;p\left(F_{test},\;F_{test}^{Prv}\right)log}}{\sum }\frac{p\left(F_{test},\;F_{test}^{Prv}\right)}{p\left(F_{test}\right)\times p\left(F_{test}^{Prv}\right)}$ is impacted significantly when obfuscation is performed. It is noticed (Figure 9) that $I\left(F_{test},\;F_{test}^{Prv}\right)\;<I\left(F_{test};F_{test}\right),$ which confirms that information content of $F_{test}$ is reduced substantially. In fact, $I\left(F_{test};F_{test}^{Prv}\right)$ is the entropy or self-information of $F_{test}$. The substantial loss information of $F_{test}^{Prv}$ due to incorporating the proposed obfuscation method, attacker’s knowledge of extracting the inference from the feature set is reduced substantially.

In the context of edge analytics, we observe that our learning model for inferential analytics generates clinically significant utility through a low dimensional feature space (total number of features = 3) while privacy-protection is through controlled Laplacian noise addition (which is with computational complexity of O(1). We find that in practice, training examples may be less in number, which may lead to weaker model generation. When training examples are continuously available at the third party, or from public databases or by self-collection, inferential model needs to be frequently re-trained. Such evolving training model invariably requires learning process development at the source or at the edge devices. Our proposed scheme and deployment architecture ensures continuous learning capability through lightweight training method. Another salient aspect that the privacy preservation approach is of utmost importance in deploying sensitive data-based analytics engine in wireless sensor network platforms to enable secure IoT applications over wireless sensor networks [29].

## 7. Conclusions

In this paper, we have demonstrated that data-driven computational methods have significant potential to infer the cardiac health condition from physiological signals like heart sound or PCG. Further, we have proposed a differential privacy based approach that obfuscates the sensitive data on-demand. Our integrated approach of computational analysis of cardiac health condition that generates inference with considerable accuracy and subsequent privacy preservation is the stepping-stone of developing the IoT driven edge cardiac health management system. Our solution has the suitability merit to be deployed in edge computing devices such that cloud infrastructure can be eliminated to ensure complete privacy protection and flexibility of frequent re-training particularly in the possible scenario of continuous learning under ever-growing availability of training signals.

We have validated our claim of significant clinical efficacy by experimenting on the publicly available, expert-annotated MIT Physionet Challenge 2016 PCG database where reliable accuracy of over 80% with good performance measures of specificity and sensitivity values are obtained. The experimental datasets being considerably large (around 3000 instances and the data collected from five different hospitals), we can safely state that our analytics method is reliable and robust. Further, we have incorporated privacy controlled information release to minimize the risk of data privacy breach. In fact, practical deployment of remote health management requires computational analysis of sensor data integrated with robust privacy protection owing to the sensitive nature of health data. In this work, we have established that integrated approach is of practically important by providing solution to efficaciously address the practical issues of implementing and deploying data-driven computational cardiac clinical analytics system. It is an integrated method to ensure privacy protected clinical utility. We have incorporated privacy protection as an integral part of clinical utility. Thus, our method enables practical acceptance to the patient community as well as to the medical community.

We require to further validating the clinical utility of our work under other relevant PCG datasets. Our future work consists of: 1. Feature space enhancement with the inclusion of few more interesting features (particularly through interacting with medical experts), 2. Deep network based (Recurrent Neural Network (RNN) or Convolution Neural Network (CNN)) based clinical analytics algorithm development for facilitating supposedly stronger training model development. The proposed algorithm is generic in nature and tailored for edge computing. We feel that in near future, bulk of the edge devices will be empowered with graphics processing unit (GPU) which will permit the deployment of deep networks like RNN and CNN.
